# O‐GlcNAc regulates anti‐fibrotic genes in lung fibroblasts through EZH2

**DOI:** 10.1111/jcmm.18191

**Published:** 2024-03-17

**Authors:** Qiuming P. Wu, Shia Vang, Jennifer Q. Zhou, Stefanie Krick, Jarrod W. Barnes, Yan Y. Sanders

**Affiliations:** ^1^ Division of Pulmonary, Allergy, and Critical Care Medicine, Department of Medicine University of Alabama at Birmingham Birmingham Alabama USA; ^2^ Department of Microbiology and Molecular Cell Biology Eastern Virginia Medical School Norfolk Virginia USA

**Keywords:** Cox2, EZH2, H3K27me3, Hmox1, lung fibroblasts, lung fibrosis, O‐GlcNAc, OGT, TGF‐ β1

## Abstract

Epigenetic modifications are involved in fibrotic diseases, such as idiopathic pulmonary fibrosis (IPF), and contribute to the silencing of anti‐fibrotic genes. H3K27me3, a key repressive histone mark, is catalysed by the methyltransferase enhancer of Zeste homologue 2 (EZH2), which is regulated by the post‐translational modification, O‐linked N‐Acetylglucosamine (O‐GlcNAc). In this study, we explored the effects of O‐GlcNAc and EZH2 on the expression of antifibrotic genes, cyclooxygenase‐2 (Cox2) and Heme Oxygenase (Homx1). The expression of Cox2 and Hmox1 was examined in primary IPF or non‐IPF lung fibroblasts with or without EZH2 inhibitor EZP6438, O‐GlcNAc transferase (OGT) inhibitor (OSMI‐1) or O‐GlcNAcase (OGA) inhibitor (thiamet G). Non‐IPF cells were also subjected to TGF‐β1 with or without OGT inhibition. The reduced expression of Cox2 and Hmox1 in IPF lung fibroblasts is restored by OGT inhibition. In non‐IPF fibroblasts, TGF‐β1 treatment reduces Cox2 and Hmox1 expression, which was restored by OGT inhibition. ChIP assays demonstrated that the association of H3K27me3 is reduced at the Cox2 and Hmox1 promoter regions following OGT or EZH2 inhibition. EZH2 levels and stability were decreased by reducing O‐GlcNAc. Our study provided a novel mechanism of O‐GlcNAc modification in regulating anti‐fibrotic genes in lung fibroblasts and in the pathogenesis of IPF.

## 1 INTRODUCTION

Fibrotic diseases contribute to approximately 45% mortality in the world.^1^ Idiopathic pulmonary fibrosis (IPF), a chronic and fatal disease, is characterized by fibroblast activation and excessive extracellular matrix deposition and has a median survival of 3–4 years.^2^ Though the pathogenesis of IPF is not totally clear, previous studies have shown that epigenetic mechanisms are likely involved in the process.^3^

Epigenetic modifications affect chromatin structure and regulate transcription.^4^ One of the best characterized repressive histone marks, H3K27me3, is catalysed by methyltransferase EZH2 (enhancer of zeste homologue 2) of the polycomb repressive complex 2 (PRC2).^5^ H3K27me3 has been documented for its involvement in fibrotic gene regulation in IPF lung fibroblasts.^6^, ^7^, ^8^ In addition, EZH2 was reported to be upregulated in IPF patients and in bleomycin‐induced lung fibrosis mouse models, and its inhibition following lung injury has been shown to attenuate fibrosis.^9^ In a study using scleroderma dermal fibroblasts, inhibition of EZH2 prevented fibrosis suggesting its potential as a therapeutic target.^5^ EZH2 has been reported as an oncogene in human cancers through epigenetically silencing tumour suppressor genes, such as RUNX3^10^ and KLF2,^11^ by increased association with H3K27me3.

The function and activity of EZH2 are regulated by post‐translational modifications such as phosphorylation, ubiquitination and O‐linked β‐N‐Acetylglucosamine (O‐GlcNAc).^12^ The O‐GlcNAc modification is transferred to proteins by the O‐GlcNAc transferase (OGT), while its removal is catalysed by O‐GlcNAcase (OGA).^13^ OGT has been documented for its involvement in metabolic processes,^14^ and IPF is considered as a metabolic related disease.^15^, ^16^ Interestingly, O‐GlcNAc has been shown to regulate EZH2 protein stability and function in breast cancer cells, which controls some tumour suppressor genes expression.^17^ However, the effects of O‐GlcNAc on EZH2 stability/expression in lung fibroblasts as well as its role in regulating anti‐fibrotic genes in IPF have not been determined.

In this study, we explored the effects of O‐GlcNAc on EZH2 and anti‐fibrotic genes in IPF. Cyclooxygenase‐2 (Cox2)^8^ and heme oxygenase‐1 (Hmox1)^18^, ^19^ genes have been documented for their anti‐fibrotic roles in lung fibrosis.^18^, ^19^ These genes have been reported to be regulated by H3K27me3.^8^, ^17^, ^20^ It is not clear whether O‐GlcNAc regulates these anti‐fibrotic genes via EZH2 mediated epigenetic mechanism in IPF lung fibroblasts. We investigated the role of O‐GlcNAc on the regulation of EZH2 and how this would affect the expression of Cox2 and Hmox1 in primary IPF fibroblasts.

## 2 MATERIALS AND METHODS

### 2.1 Cell culture and treatments

Primary human IPF and non‐IPF fibroblasts were obtained from the University of Alabama at Birmingham (UAB) Tissue Procurement Facility, derived from de‐identified lung tissues, which protocol is approved by the UAB Institutional Review Board. The diagnosis of IPF was made by a multidisciplinary approach according to American Thoracic Society/European Respiratory Society guidelines.^21^ One of the non‐IPF cell lines, IMR90, was purchased from ATCC. The primary cells were all used before Passage 5. All cells were kept in Dulbecco's modified Eagle's medium (DMEM, Life Technologies, Grand Island, NY) with 10% fetal bovine serum (FBS, Life Technologies), 100 units/mL penicillin, 100 μg/mL streptomycin, 1.25 μg/mL amphotericin B and 2 mM L‐glutamine (G1251, Sigma) at 5% CO_2_ at 37°C. Inhibitors were added directly in the cell culture media for 24 h as follows: EZH2 inhibitor EZP6438 at 5 μM, OGT inhibitor OSMI‐1 at 25 μM and O‐GlcNAcase (OGA) inhibitor Thiamet G at 25 nM. For non‐IPF cells with TGF‐β1 addition, the indicated inhibitor was added 2 h prior to adding TGF‐β1 at 2 ng/mL. The cells were harvested for various assays after 24 h or as indicated.

### 2.2 RNA extraction and quantitative real‐time RT‐PCR

RNA was extracted with the RNeasy Mini Kit (Qiagen, Valencia, CA), and reverse transcribed into cDNA using a cDNA synthesis kit (Takara Bio, Mountain View, CA). Quantitative real‐time reverse transcription‐PCR was performed in triplicates and normalized to β‐actin by using the 2^−ΔΔCT^ method.^22^ mRNA levels of Cox2 and Hmox1 were examined using the quantitative real‐time PCR with primers as follows: Cox2: F: 5′‐CCGGGTACAATCGCACTTAT‐3′ and R: 5′‐ GGCGCTCAGCCATACAG‐3′; Hmox1: F: 5′‐CCAGGCAGAGAATGCTGAGTTC‐3′ and R: 5′‐AAGACTGGGCTCTCCTTGTTGC‐3′; and β‐actin: F: 5′‐ TGCTATCCAGGCTGTGCTAT −3′, and R: 5′‐ AGTCCATCACGATGCCAGT‐3′.

### 2.3 Protein extraction and Immunoblotting

Cells were washed in cold PBS and lysed with 2× SDS reducing buffer with protease inhibitors to make whole cell lysates. The protein concentration was measured by a Micro BCA Protein Assay kit (Thermo Scientific). Lysates were then subjected to SDS‐PAGE; and immunoblotting was performed as described before.^22^ Densitometric analysis was done using ImageJ software. Antibodies against EZH2 (Cell signalling, Cat# 5246), H3K27Me3 (Cell Signalling, Cat# 9733), Cox2 (Abcam, Cat# ab15191), Hmox1 (Cell signalling, Cat# 5853) were used, and the signals were detected using an enhanced chemiluminescence system and imaged with an Amersham Biosciences 600 Imager (GE Healthcare).

### 2.4 Chromatin immunoprecipitation assays

Chromatin immunoprecipitation (ChIP) assays were performed as per the manufacturer's protocol (ab500, Abcam, Cambridge, MA) with minor modifications.^23^, ^24^ The antibody against H3K27Me3 (cat#39155) used for ChIP assays was obtained from Active Motif (Carlsbad, CA). For ChIP assays, the following primers were used: Cox2, F: 5′‐ TGACTTCCTCGACCCTCTAA‐3′, and R: 5′‐ GGACACTTGGCTTCCTCTC‐3′; Hmox1, F: 5′‐ TGAGGAGGCAAGCAGTC‐3′, and R: 5′‐ GTGGGCAACATCAGGAACTTA‐3′. A pull‐down with IgG served as the negative control. ChIP‐DNA was amplified by real‐time PCR, using SYBR® Green PCR Master Mix (Life Technologies). Results were normalized to input DNA.

### 2.5 Immunoprecipitation

Primary lung fibroblasts were grown to 70% confluency and treated with either the OGT or OGA inhibitors (as above), trypsinized and collected for immunoprecipitation (IP) as previously described^25^ with some modifications. Briefly, 1 g of protein cell lysates were pre‐cleared with a 60 μL mixed slurry of unblocked protein L agarose beads (Santa Cruz, CA, USA) by continuous rocking for 1 h at 4°C to remove any non‐specific binding. Then, the pre‐cleared cell lysates were subjected to antisera against O‐GlcNAc [CTD110.6 antibody (IgM); 4 μg/1 g protein; Biolegend, San Diego, CA, USA] that was added overnight at 4°C. The O‐GlcNAc specific‐antibody complexes were purified from lysates using blocked protein L agarose (Santa Cruz Biotech, Dallas, TX, USA) followed by four washes using RIPA buffer (Cell Signalling, MA, USA), two washes with 1.0% Triton‐X 100 in 20 mM Tris pH 6.8 and three washes with a final wash buffer containing 20 mM Tris–HCl pH 6.8. Partially purified O‐GlcNAc modified proteins were eluded from the protein L beads by boiling in 4X Laemmli buffer (Bio‐Rad, USA) and subjected to SDS‐PAGE. To determine whether EZH2 is modified by O‐GlcNAc, immunoblot analysis of EZH2 was performed using antisera against EZH2 (Cell signalling, Cat# 5246). In all cases, 5.0% of the whole tissue lysate (before IP) was set aside as a western blot (WB) input control, to ensure equal loading, and probed with EZH2.

### 2.6 Protein stability assays

Primary lung fibroblasts were grown to 80% confluency and then treated with cycloheximide (CHX; 50 μg/mL) for 0, 8, 16 and 24 h in the presence or absence of ThG or OSMI‐1. Protein extraction of whole cell lysates were prepared in 2× SDS reducing buffer with protease inhibitors. The protein concentration was measured by a Micro BCA Protein Assay kit (Thermo Scientific). Lysates were then subjected to SDS‐PAGE and western immunoblotting with antibodies against EZH2 (Cell signalling, Cat# 5246), or β‐actin for loading control. The blots were imaged with an Amersham Biosciences 600 Imager (GE Healthcare) and densitometry analysis was done using ImageJ software.

### 2.7 Statistical analysis

Data are presented as the mean ± standard error (SE). All data were statistically analysed using GraphPad Prism 5.0 (La Jolla, CA). One‐way ANOVA was used to compare between multiple groups; comparison between two groups was with Student's *t*‐test. A *p‐*value of less than 0.05 was considered to be statistically significant.

## 3 RESULTS

### 3.1 Expression of Cox2 and Hmox1 in primary human IPF lung fibroblasts is upregulated following inhibition of OGT

**FIGURE 1:**
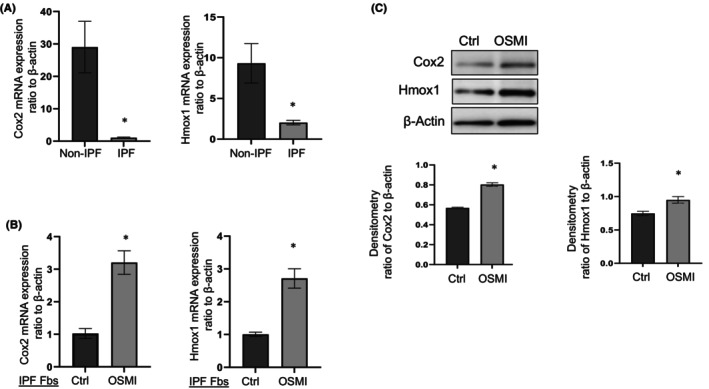
Cox2 and Hmox1 expression in non‐IPF and IPF primary fibroblasts with or without OGT inhibitor OSMI‐1. (A) Expression of Cox2 and Hmox1 at mRNA level in non‐IPF and IPF primary lung fibroblasts, measured by quantitative real‐time RT‐PCR, normalized to β‐Actin. Results are averages of three or four different primary lung fibroblasts with at least three independent repeats of each. (B) mRNA level expression of Cox2 and Hmox1 in IPF primary lung fibroblasts treated with vehicle control (Ctrl) or OGT inhibitor OSMI‐1 at 25 μM for 24 h. (C) Protein level expression of Cox2 and Hmox1 in IPF fibroblasts treated with OGT inhibitor OSMI as in (B). β‐Actin is the loading control. Lower panels are the densitometry of Cox2 or Hmox1 normalized to β‐Actin. Results are mean ± SE of at least three independent experiments. *p < 0.05, IPF versus non‐IPF, or OSMI treated versus vehicle.

Previous studies in IPF reported reduced expression of the anti‐fibrotic genes Cox2 and Hmox1.^8^, ^18^, ^26^ First, we re‐confirmed the expression levels of these two genes in primary non‐IPF and IPF lung fibroblasts. Cox2 and Hmox1 were significantly reduced at the mRNA level in primary IPF fibroblasts compared to non‐IPF controls (Figure 1A).

Next, we determined the effects of blocking OGT on Cox2 and Hmox1 expression in IPF lung fibroblasts. Following OGT inhibition, Cox2 and Hmox1 expression at both mRNA and protein levels were significantly increased (Figure 1B,C). These findings suggest that Cox2 and Hmox1 are upregulated or restored following OGT inhibition in IPF fibroblasts.

### 3.2 Inhibition of OGT activity blocks TGF‐β1 induced downregulation of Cox2 and Hmox1 in human lung fibroblasts

**FIGURE 2:**
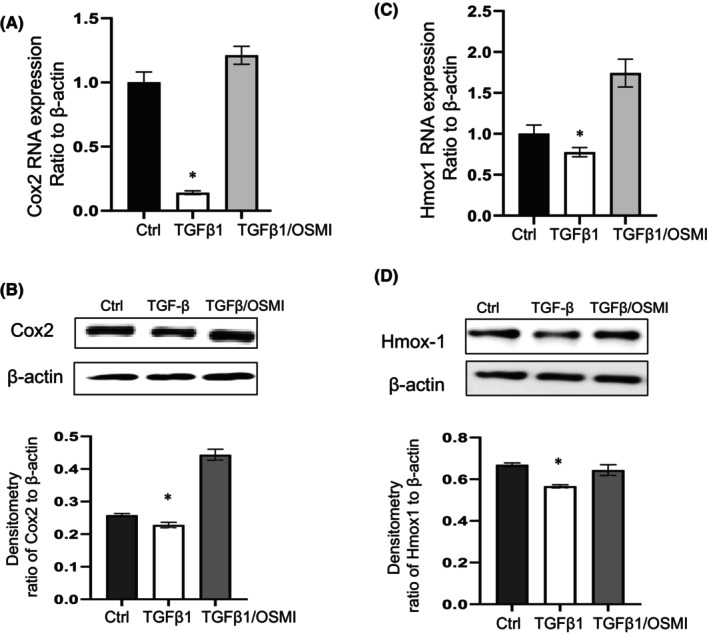
Expression of Cox2 and Hmox1 in non‐IPF fibroblasts with TGF‐β1 with or without pre‐treatment of OGT inhibitor OSMI‐1. Primary non‐IPF fibroblasts were treated with TGF‐β1 at 2 ng/mL for 24 h, the group with OSMI‐1 treatment were added 2 h before adding TGF‐β1 at 25 μM. Cox2 expression at mRNA (A) and protein (B) or Hmox1 at mRNA (C) or protein (D) levels are examined by quantitative real‐time RT‐PCR, normalized to β‐Actin (A, C), or by western blots (B, D), with β‐Actin as the loading control. Densitometric analysis of WB as shown in B or D normalized to β‐Actin. Results are mean ± SE of at least three independent experiments. *p < 0.05 versus vehicle control or TGF‐β1 with OSMI.

Transforming growth factor‐β1 (TGF‐β1) is a well‐documented pro‐fibrotic cytokine that mediates fibroblast differentiation,^27^ and is regarded as one of the master regulator of lung fibrosis.^28^ TGF‐β1 has been reported to activate lung fibroblast, while reducing Cox2^20^ and Hmox1 expression.^19^ Since reducing O‐GlcNAc via OGT inhibition upregulated these anti‐fibrotic genes in IPF fibroblasts, we further examined whether OGT inhibition would block TGF‐β1‐induced downregulation of Cox2 and Hmox1. Non‐IPF fibroblasts were treated with OGT inhibitor for 2 h before adding TGF‐β1 at 2 ng/mL for 24 h. TGF‐β1 induced downregulation of Cox2 and Hmox1, and the pre‐treatment of OGT inhibitor attenuated the downregulation of Cox2 (Figure 2A, B) and Hmox1 (Figure 2C, D) at the mRNA and protein levels. Therefore, our data suggest that OGT inhibition blocks TGF‐β1 induced downregulation of Cox2 and Hmox1 in lung fibroblasts.

### 3.3 Reducing O‐GlcNAc levels decreases the association of the repressive histone H3K27me3 at the Cox2 and Hmox1 promoter regions

**FIGURE 3:**
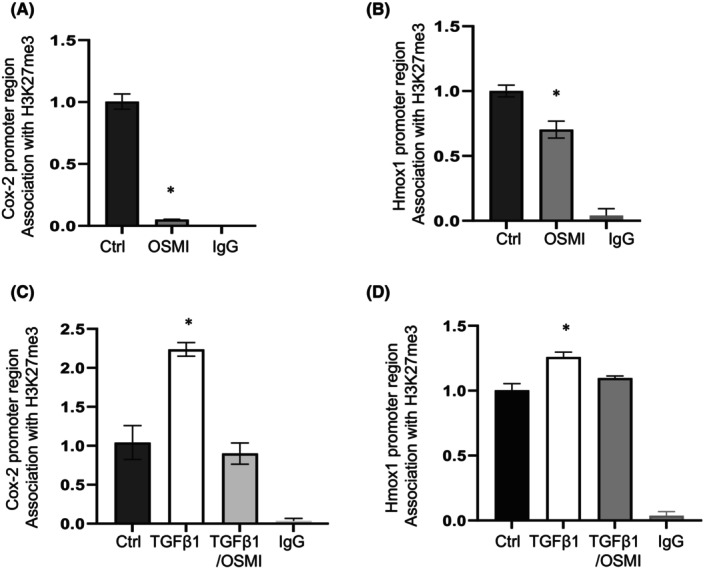
Cox2 and Hmox1 promoter region association with H3K27me3. (A, B) IPF fibroblasts were treated with vehicle control or OSMI‐1 at 25 μM for 24 h, before subjected to ChIP assays. (C, D) Non‐IPF fibroblasts were treated with or without OSMI‐1 (25 μM) 2 h before adding in TGF‐β1 at 2 ng/mL for 24 h for ChIP assays. The Cox2 (A, C) or Hmox1 (B, D) promoter region association with H3K27me3 was analysed by ChIP assays with primers listed in the methods section. DNA was immunoprecipitated with specific antibody against H3K27me3, and IgG was used as a negative control. Quantitative PCR data were analysed using 2^−ΔΔCt^ method, and results were normalized to input DNA expressed as fold change relative to vehicle control (Ctrl). The values are expressed as mean ± SE from average of three independent experiments from one cell line. *p < 0.05, versus Control or TGF‐β1 with OSMI pretreatment.

Cox2 and Hmox1 have been shown to be regulated by the repressive histone H3K27me3.^8^, ^17^ We therefore investigated whether increased expression of Cox2 and Hmox1 is regulated by H3K27me3 following OGT inhibition. ChIP assays were carried out with an anti‐H3K27me3 antibody pull‐down, and the associations of Cox2 and Hmox1 promoter regions with H3K27me3 were examined. In IPF lung fibroblasts treated with OSMI‐1, we observed significantly depleted H3K27me3 at the promoter region of Cox2 and Hmox1 (Figure 3A,B), which corresponded to the upregulated mRNA expression of these genes (Figure 1B). In response to TGF‐β1, lung fibroblasts exhibited a significantly enriched association of H3K27me3 at the Cox2 (Figure 3C) and Hmox1 (Figure 3D) promoter regions, corresponding to downregulation of their mRNA by TGF‐β1 (Figure 2A, C). However, OGT inhibition significantly depleted the TGF‐β1 induced association of H3K27me3 with the Cox2 and Hmox1 promoter regions (Figure 3C, D), which blocked the mRNA downregulation (Figure 2A, B). Overall, our study suggests that the H3K27me3 repressive mark is involved in the regulation of Cox2 and Hmox1 mRNA expression following OGT inhibition.

### 3.4 Pharmacological inhibition of the H3K27me3 methyltransferase, EZH2, upregulates Cox2 and Hmox1 expression in a similar fashion as OGT inhibition

**FIGURE 4:**
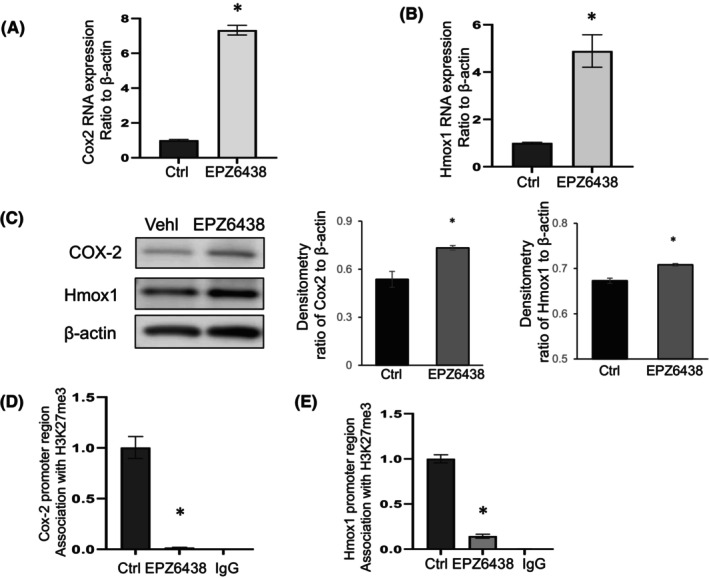
Cox2 and Hmox1 expression change in response to EZH2 inhibitor EZP6438. IPF fibroblasts were treated with EZH2 inhibitor EZP6438 at 5 μM for 24 h. Then the cells were collected for further analysis of RNA, protein level expression and ChIP assays. (A, B) RNA level expression of Cox2 (A) and Hmox1 (B) was analysed with quantitative real‐time RT‐PCR, with ratio to β‐Actin. (C) Protein level expression of Cox2 and Hmox1 were examined by western blots, β‐Actin is the loading control. Densitometric analysis of WB as shown in C of Cox2 or Hmox1 normalized to β‐Actin. (D, E) DNA was immunoprecipitated with specific antibody against H3K27me3, and IgG was used as negative control. qPCR data were analysed using 2^−ΔΔCt^ method, and results were normalized to input DNA expressed as fold change relative to vehicle control (Ctrl). Results are mean ± SE of at least three independent experiments. *p < 0.05 versus vehicle control.

To confirm whether changes in H3K27me3 association at the promoter regions of Cox2 and Hmox1 would impact gene expression, IPF fibroblasts were treated with an EZH2 inhibitor (EPZ6438).^29^ First, the activity of the inhibitor was confirmed and showed significantly reduced H3K27me3 levels in the fibroblasts (Figure S1). Analysis of the expression of Cox2 and Hmox1 suggested upregulation at the mRNA (Figure 4A, B) and protein (Figure 4C) levels. To further check whether EZH2 inhibition depleted the association of H3K27me3 at the Cox2 and Hmox1 promoter regions, we carried out ChIP assays with anti‐H3K27me3 antibody pull‐down. Following EZH2 inhibition, H3K27me3 was significantly depleted at the promoter regions of these genes (Figure 4D, E). Overall, our data suggest that EZH2 inhibition reduces the association of H3K27me3 at the promoter regions of Cox2 and Hmox1 resulting in the upregulation of gene expression in IPF lung fibroblasts.

### 3.5 EZH2 levels are controlled and stabilized by the O‐GlcNAc modification in human lung fibroblasts

**FIGURE 5:**
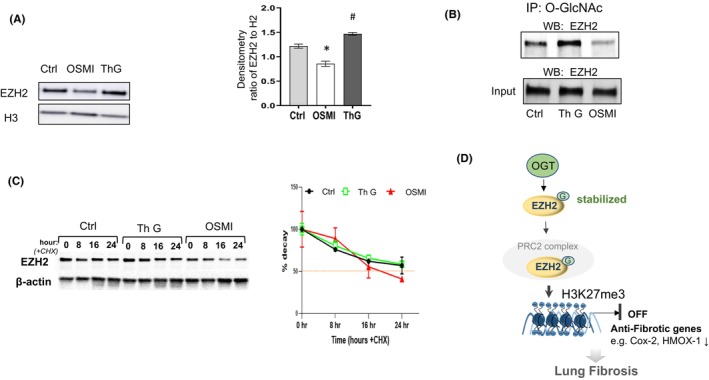
EZH2 in lung fibroblasts is regulated by the O‐GlcNAc axis. (A) A representative WB of EZH2 levels in primary IPF lung fibroblasts treated with OSMI at 25 μM or Thiamet G (ThG) at 25 nM for 24 h. Nuclear extracts were collected for WB, H3 is the loading control. Right panel: densitometry of EZH2 ratio to H3, *n* = 3 of experiment repeats. More treated cell lines in (Figure S2). (B) Lung fibroblasts were treated with ThG or OSMI‐1 as above in (A), then the nuclear protein was immunoprecipitated (IP) with O‐GlcNAc antibody and subjected to WB as done previously.^25^ Following IP with O‐GlcNAc, partially enriched samples were Western blotted for EZH2 (top panel). As a loading control, 5.0% of the whole tissue lysate (before IP) was set aside (input, *bottom panel*), to ensure equal loading, and probed with EZH2. (C) WB and graph of % decay of EZH2 following cycloheximide (CHX, 50 μg/mL) treatment with ThG (25 nM) or OSMI‐1 (25 μM) and for 0, 8, 16 and 24 h. Whole cell lysate were collected and subjected to WB and densitometry at the time points indicated, normalized to CHX time at 0 h. (D) Schematic diagram of possible mechanisms of the findings from this study. The reduced expression of anti‐fibrotic genes, Cox2 and Hmox1, are partially due to the increased binding of repressive histone H3K27me3 to their promoter region, which is likely mediated through EZH2 that is stabilized via O‐GlcNAc by OGT in lung fibroblasts.

Similar effects from EZH2 inhibition (Figure 4) and OGT inhibition (Figures 1 and 3) on the upregulation of Cox2 and Hmox1 were observed in lung fibroblasts. Therefore, we wanted to examine whether the upregulation is mediated through O‐GlcNAc modification of EZH2 in lung fibroblasts. IPF fibroblasts were treated with either OSMI‐1 or Thiamet G for 24 h. We observed reduced EZH2 expression in cells following OGT inhibition (reduced O‐GlcNAc), while increased EZH2 was observed following OGA inhibition (increased O‐GlcNAc) (Figure 5A) suggesting that the levels of EZH2 are regulated by O‐GlcNAc in lung fibroblasts. To determine whether EZH2 is directly modified by O‐GlcNAc, we performed immunoprecipitation (IP) using the O‐GlcNAc antibody (CTD 110.6). The lung fibroblasts lysates were subjected to immunoprecipitation with antibody against O‐GlcNAc, which specifically recognizes O‐GlcNAc. An antibody specific to EZH2 was used to check whether EZH2 was modified by O‐GlcNAc. By WB, EZH2 was present in equivalent amounts in all the input samples. Following O‐GlcNAc pull‐down, EZH2 was observed to be modified by O‐GlcNAc, which showed increased amount with OGA inhibition, whereas the amount of O‐GlcNAc was reduced on EZH2 following OGT inhibition (Figure 5B). These data demonstrate that EZH2 is O‐GlcNAc modified in lung fibroblasts.

Previous reports in breast cancer cells have shown that the O‐GlcNAc modification regulates EZH2 protein stability and function.^17^ To examine whether O‐GlcNAc controls EZH2 protein stability in lung fibroblasts, we treated the cells with cycloheximide to inhibit protein synthesis and monitored the remaining EZH2 protein levels for up to 24 h. Our data demonstrate that EZH2 levels decrease over time in the non‐treated and OGA inhibitor treated groups; however, the levels of EZH2 were much lower following OGT inhibition (Figure 5C). Taken together, these experiments demonstrate that reducing O‐GlcNAc levels decreases EZH2 stability in lung fibroblasts. This most likely contributes to the reduced repressive H3K27me3 mark at the promoter regions of Cox2 and HMOX1 and resulting upregulation of these genes.

## 4 DISSCUSSION

In this study, we show a mechanism for the regulation of anti‐fibrotic genes in lung fibroblasts. We demonstrate that the stability of EZH2 is decreased by reducing the O‐GlcNAc modification of the protein, which in turn reduces H3K27me3 enrichment at the promoter regions of Cox2 and Hmox1. The depleted enrichment results in upregulated expression of these anti‐fibrotic genes in fibrotic lung fibroblasts with OGT inhibition (Figure 5D).

EZH2 is the methyltransferase of histone H3K27me3, which is a well‐known marker associated with transcriptional repression.^30^ EZH2 is the catalytic subunit of polycomb‐repressive complex 2 (PRC2) that represses transcription.^30^ EZH2 has a wide range of functions, including regulation of cell cycle progress, autophagy, apoptosis, DNA damage repair and cellular senescence.^31^ Besides its function as methyltransferase, EZH2 can activate transcription that is independent of its role as methyltransferase.^32^ EZH2 is involved in many diseases, including fibrosis. Increased EZH2 is reported in fibrotic diseases, such as pulmonary fibrosis,^7^, ^9^ renal fibrosis,^33^ liver fibrosis^34^, ^35^ and scleroderma^5^; and inhibition of EZH2 showed improved fibrotic conditions.

EZH2 regulates transcription via H3K27me3 mediated gene repression.^36^ In a study in gastric cancer, EZH2 was shown to bind to the p21 promoter region and mediate H3K27me3 modification, a process that resulted in silencing of p21 and induction of cancer cell proliferation.^36^ In another study in lymphoma, EZH2 mutation led to increased enzymatic activity that promoted H3K27me3 and suppressed gene expression.^37^ Indeed, many types of mechanisms have been reported in the regulation of EZH2 expression, stability and enzymatic activity.^17^, ^38^, ^39^, ^40^, ^41^ Post‐translational modifications, including O‐GlcNAc, have been investigated for their role in regulating EZH2 stability.^42^

O‐GlcNAcylation is reversible and dynamic, catalysed by OGT and OGA.^43^ O‐GlcNAcylation plays a key role in many cellular processes, including transcription and cellular signalling and its dysregulation has been observed in many diseases.^44^ An early study in breast cancer has shown that O‐GlcNAc at Ser‐75 stabilized EZH2, and facilitated H3K27me3 on target genes.^17^ The same group later reported more sites of O‐GlcNAc modification on EZH2, which controlled different biological functions.^41^ Another study demonstrated that EZH2 interacted with OGT in a cell type‐specific way to regulate downstream target gene expression.^45^ To our knowledge, the O‐GlcNAc regulation of EZH2/gene expression in lung fibrosis has not been examined; therefore, we explored whether this regulatory mechanism applied to lung fibroblasts and would contribute to the pathogenesis of lung fibrosis.

In our current study, we showed that EZH2 was modified by O‐GlcNAc, which regulated EZH2 protein level expression and stability. In addition, the loss of stability of EZH2 had downstream consequences on H3K27me3 association with the promoter region of the anti‐fibrotic genes, Cox2 and Hmox1, in lung fibroblasts, which further altered the expression of these genes. We picked these two anti‐fibrotic genes as previous reports indicated their expression was partially regulated by H3K27me3.^8^, ^17^ Our data showed that the reduced expression of these genes that were observed in IPF fibroblasts was upregulated following OGT blockade. We believe that the reduction in O‐GlcNAc levels by OGT inhibition diminished the stability of EZH2, which reduced the repressive H3K27me3 mark at these genes' promoter regions. However, OGT inhibition may also affect other processes besides EZH2/H3K27me3 to regulate gene expression. Therefore, we confirmed the role of EZH2/H3K27me3 regulation of gene expression through the inhibition of EZH2, in which we obtained a similar pattern of gene regulation on Cox2 and Hmox1.

Besides EZH2, OGT has been shown to interact with other transcriptional regulators, including components of the polycomb group (PcG), whose main complexes are polycomb repressive Complex 1 and 2 (PRC1 and 2).^46^, ^47^ It is possible that a change in association of H3K27me3 at the Cox2 and Hmox1 promoter region may be an overall consequence of OGT inhibition. Among the PcG proteins that interact with OGT, EZH2 happens to be a known direct target of OGT and the O‐GlcNAc modification has been shown to be essential for EZH2 stability.^41^ This finding is consistent with our study in lung fibroblasts. Other transcriptional repression complexes, such as mSin3A, have been shown to associate with histone deacetylases (HDACs) 1 and 2, and have been reported to interact with OGT to repress transcription.^48^ Although Cox2 and Hmox1 have been shown to be regulated by HDAC1 or HDAC2,^49^, ^50^ it is possible that OGT inhibition may also alter HDACs and contribute to the expression change of these two genes. However, our focus of the current study was to explore the effects of OGT on histone methyltransferase, EZH2, and its downstream targets. The effects on HDACs as a possible mechanism in lung fibroblasts warrant further investigation.

O‐GlcNAc has been shown to regulate histone modifications that control many cellular processes.^13^ We only examined the changes of H3K27me3 at Cox2 and Hmox1 promoter regions, as that directly contribute to the regulation of these genes. Although we only studied two fibrotic‐related gene expression changes in response to O‐GlcNAc modulation, studies are currently underway to explore other genes (pro‐ and anti‐fibrotic) that are regulated by O‐GlcNAc‐mediated epigenetic mechanisms. Nevertheless, our results demonstrate that O‐GlcNAc regulates gene expression in lung fibroblasts through EZH2/H3K27me3, indicating that O‐GlcNAc controlled epigenetic programming of histone modifications may be a possible therapeutic strategy in fibrotic diseases.

## AUTHOR CONTRIBUTIONS

**Qiuming P. Wu:** Data curation (lead); formal analysis (lead); investigation (equal); writing – original draft (equal). **Shia Vang:** Data curation (supporting); formal analysis (equal); writing – review and editing (supporting). **Jennifer Q. Zhou:** Data curation (equal); formal analysis (supporting); validation (lead); writing – review and editing (supporting). **Stefanie Krick:** Conceptualization (supporting); formal analysis (equal); writing – review and editing (supporting). **Jarrod W. Barnes:** Conceptualization (equal); formal analysis (equal); funding acquisition (equal); project administration (equal); writing – original draft (equal); writing – review and editing (equal). **Yan Y. Sanders:** Conceptualization (lead); formal analysis (lead); funding acquisition (lead); project administration (lead); writing – original draft (lead); writing – review and editing (equal).

## ACKNOWLEGEMENTS

The authors thank Dr. E Scott Helton at UAB for his technical support for this project. This work was supported by the National Institutes Health grants, R01HL152246 to JWB, R01AG050567 and 1R01HL151702 to YYS.

## CONFLICT OF INTEREST STATEMENT

The authors confirm that there are no conflicts of interest.

## Supporting information


Data S1.

## Data Availability

The data that supports the findings of this study are available in the text and supplementary material of this article.
