# Supplementary material for: O‐GlcNAc regulates anti‐fibrotic genes in lung fibroblasts through EZH2
Source: J Cell Mol Med. 2024 Mar 17;28(7):e18191. doi: 10.1111/jcmm.18191 (PMC10945079; doi:10.1111/jcmm.18191)
Supplement: Supplementary file 1 — Data S1. [file JCMM-28-e18191-s001.pdf]

O-GlcNAc Regulates Anti-Fibrotic Genes in Lung Fibroblasts through EZH2

Qiuming P Wu, Shia Vang, Jennifer Q Zhou, E Scott Helton, Stefanie Krick, Jarrod W Barnes, Yan Y Sanders

Online supplementary material

Supplementary Figure 1S

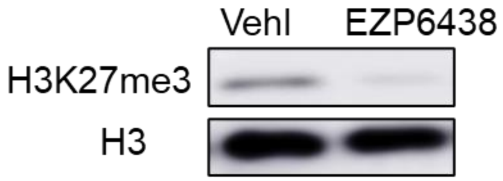

Supplementary Figure 1S: IPF fibroblasts were treated with EZH2 inhibitor EZP6438 at 5µM for 24h. Nuclear extracts were subjected to western blots, with anti-bodies against H3K27me3 and H3. H3 is loading control.

Supplementary Figure 2S

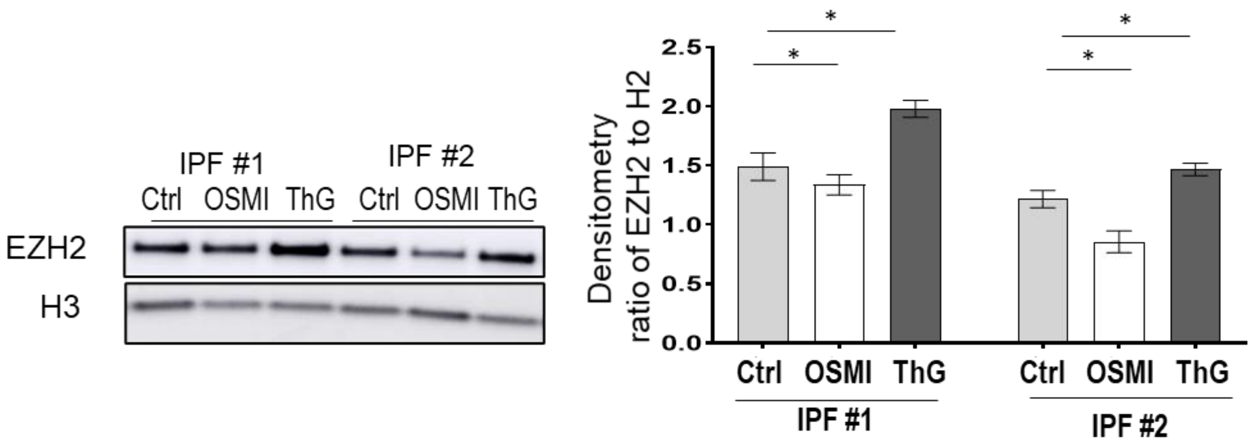

Supplementary Figure 2S: Additional IPF cell lines treated as in Figure 5A. Western blots of EZH2 levels lung fibroblasts treated with OSMI at 25µM or Thiamet G (ThG) at 25nM for 24 h. Nuclear extracts were collected for WB, H3 is the loading control. Right panel: densitometry of EZH2 ratio to H3, n=3 of experiment repeats.
